# Macroscopic emulsion drops formed through limited coalescence

**DOI:** 10.1140/epje/s10189-026-00582-y

**Published:** 2026-05-12

**Authors:** D. Langevin

**Affiliations:** Laboratoire de Physique Des Solides, CNRS-Université Paris Saclay, Orsay, France

## Abstract

This short paper is a perspective article related to the seminal paper: S. Arditty, C. P. Whitby, B. P. Binks, V. Schmitt & F. Leal-Calderon “Some general features of limited coalescence in solid-stabilized emulsions” published in 2003 (Arditty et al. in Eur. Phys. J. E 12:355, 2003). I will first briefly recall the work described in this paper and discuss some of the developments that followed its publication. This will include comparisons with different coalescence mechanisms, together with comparisons with surfactant-stabilized emulsions and with particle-stabilized foams. I will end with a few comments on the state of the field of research and on future directions.

**Graphic abstract:**

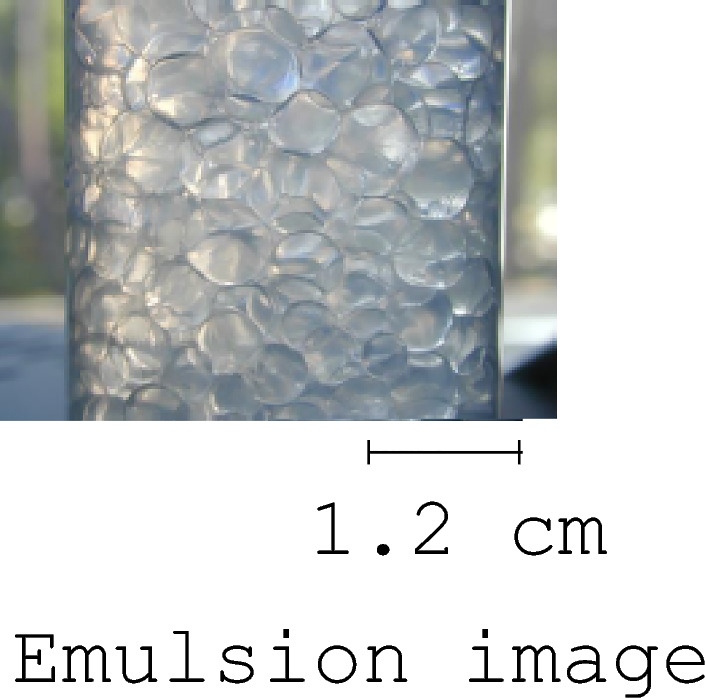

## Retrospective

This short paper is a perspective article related to the seminal paper: S. Arditty, C. P. Whitby, B. P. Binks, V. Schmitt & F. Leal-Calderon “Some general features of limited coalescence in solid-stabilized emulsions” published in 2003 in the European Physical Journal [1]. It will be referred to as the EPJE paper in the following.

Emulsions stabilized by colloidal particles, also called *Pickering* emulsions, are much more stable than emulsions stabilized by surfactants, because the particles form a thick rigid layer at the surface of the emulsion drops [2]. Although this advantage was already pointed out by Pickering in 1907, these emulsions were largely ignored for a long period until a renewal of interest arose at the end of the 1990s. During this time gap however, some Pickering emulsions were investigated without acknowledging them as such, especially in the field of food emulsions where small fat crystals often adsorb at drop surfaces. The origin of the remarkable stability of Pickering emulsions began to be investigated during the last 25 years. An important step forward was the use of silica particles of the same size distribution but increasing silane coating extent which were synthesized under the lead of Bernard Binks, one of the authors of the EPJE paper. Binks could then explore the role of particle hydrophobicity. He showed that when the contact angle *θ* between the particles and the oil–water interface (measured through the aqueous phase) is less than 90°, oil in water (O/W) emulsions are formed, whereas when *θ* is larger than 90°, water in oil (W/O) emulsions are formed. When *θ* < 90°, the particles can be dispersed in water, whereas when *θ* > 90°, they can be dispersed in oil; hence, the emulsion type follows, as with surfactants, the well-known Bancroft rule [3]. Véronique Schmitt and Fernando Leal-Calderon, also authors of the EPJE paper, reported later similar correlations with the contact angle that they measured directly at the oil–water interface, using surface-active lipophilic core–hydrophilic shell latex particles [4].

**Fig. 1:**
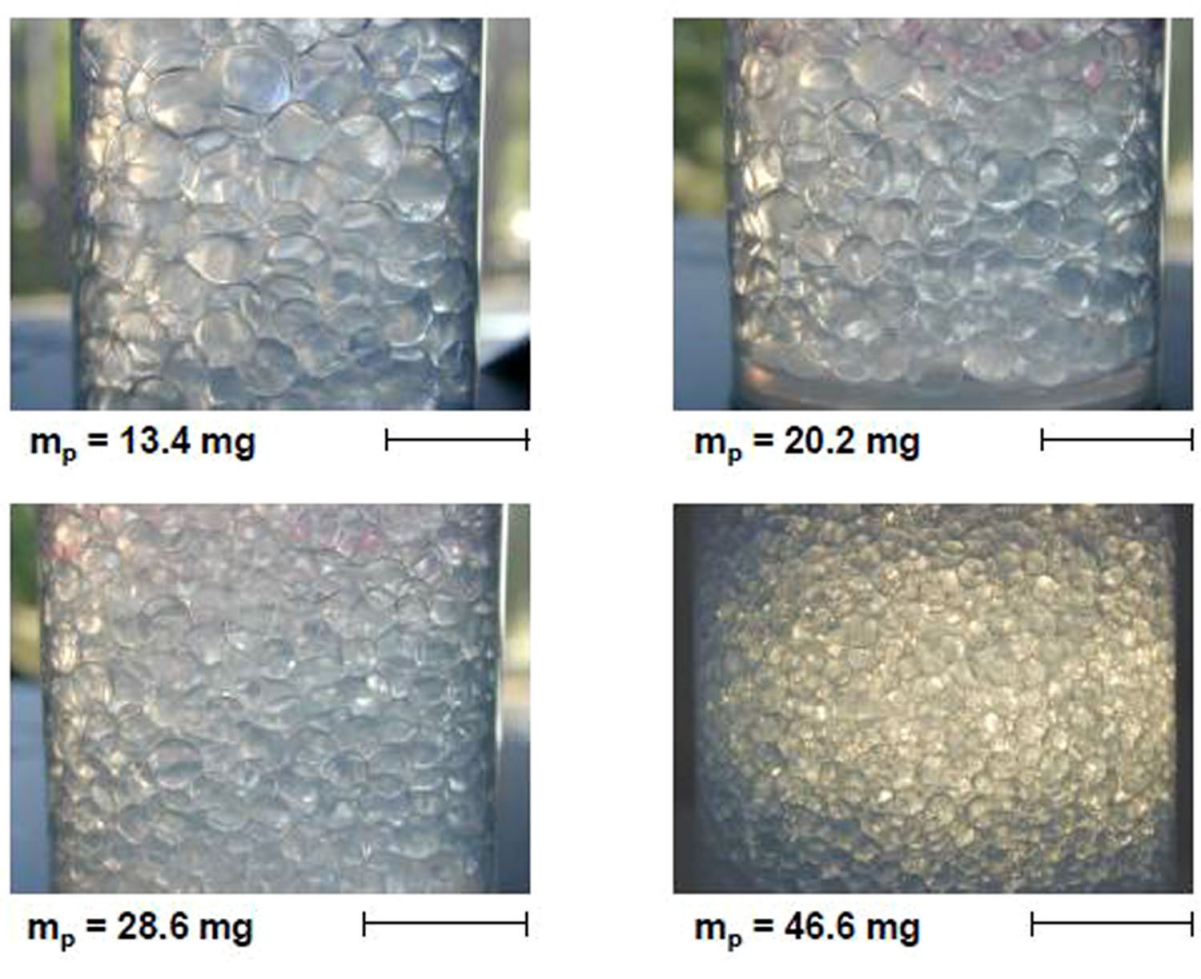
Images of O/W emulsions at long times containing 80 wt.% of 350 cP PDMS obtained for different masses of partially hydrophobic silica particles, *m*_p_ (given), the mass of emulsions being 50g. Scale bar = 1.2 cm. Color image kindly provided by Véronique Schmitt

In the EPJE paper, the authors used partially hydrophobic silica particles to study O/W and W/O emulsions. They chose to use an amount of particles initially insufficient to fully cover the drops (particle-poor regime). Emulsions were made by shaking a mixture of equal volumes of oil and water phases, after which small amounts of dispersed phase were added, in order to obtain concentrated emulsions (fraction of disperse phase ~ 80%). After preparation, the oil drops coalesce and grow reaching sizes large enough to be observed by eye as shown in Fig. 1. Coalescence stopped when the oil–water interface was sufficiently well covered by particles. This limited coalescence phenomenon leads to a final drop size governed by the initial amount of particles. The emulsion images reveal a structure similar to foams, illustrating the similarities between the two types of dispersions, which are both stabilized by surface-active agents. In the EPJE paper, these concentrated emulsions are called *bi-liquid foams*. Note that concentrated emulsions are also called *high internal phase emulsions* (HIPE).

The authors quotes that their emulsions can be stored at rest for months without any structural evolution. Even after several months, oil droplets at the top of some o/w emulsions are only slightly deformed, *i.e.*, not strongly faceted, as long as the droplet diameter does not exceed 1 cm. This is because capillary forces are much larger than gravity forces, especially in view of the small difference between the silicone oil used and water (0.04 g/cm^3^). A precise calculation of the change in dispersed volume fraction with height in concentrated emulsions using ref. [5] confirms the observation.

During the growth of oil drops, once stirring is stopped, the total interfacial area between oil and water is progressively reduced and since the particles are irreversibly adsorbed, the degree of drop surface coverage increases. Drop sizes were measured and shown to increase with time until the drop surface coverage reaches values of the order of 200–300 mg/m^2^, independently of the total amount of particles. In other words, the larger this amount, the smaller the drops (see Fig. 1).

It was noted by the authors that the limit coverage is about 10 times larger than the coverage by a dense particle monolayer, suggesting that the surface layer is made of particle aggregates. Interestingly, when the emulsions are made using a high-speed mixer (Ultra-Turrax homogeneizer), the maximum coverage is smaller and corresponds to a particle monolayer, probably because the aggregates are broken in the high shear produced by the emulsification machine. It is worth mentioning that some Pickering emulsions contain drops with a still smaller surface coverage [6].

Another interesting observation is the narrow drop size distribution (polydispersity index, as defined by the authors, of the order of 10%), despite the turbulent flow present during emulsion preparation.

This behavior is quite different from that of surfactant-stabilized emulsions. Concentrated emulsions with drops initially well covered remain stable for a certain time, after which coalescence begins and the polydispersity increases rapidly [7]. This can be explained by assuming a constant frequency of coalescence per unit area *ω *[8], a hypothesis confirmed recently with experiments with foams [9]. Neighboring drops being in permanent contact, larger drops grow faster because they possess a larger contact area with their neighbors. Note that in these emulsions, the drops were initially well covered.

In the Pickering emulsions studied, *ω* is not constant because the surface coverage changes with time. Dilute Pickering emulsions were studied by Wiley and later by Whitesides and Ross, who also observed a limited coalescence process ending with rather monodisperse drops. Wiley accounted for the final size of the drops [10], whereas Whitesides and Ross accounted for the small polydispersity by assuming that *ω* is proportional to the uncovered surface fraction of the drops [11]. In the EPJE paper, *ω *was estimated and indeed found to decrease with time. The fact that *ω* is not constant, explains why the coalescence process is quite different from that of the surfactant-stabilized emulsions with initially well-covered drops.

Limited coalescence was observed with surfactant-stabilized emulsions in a different context by Cabane and coworkers [12]. They distinguished two regimes during the emulsification process, a surfactant-rich regime, in which the drop size is determined by the emulsifying device and a surfactant-poor regime in which there is not enough surfactant to cover the drops that coalesce until they reach a suitable surface coverage. For the surfactant used, sodium dodecyl sulfate (SDS), the coverage can be significantly less than that of a dense monolayer (~ 10 mg/m^2^), by a factor up to 100. This is because the surfactant is ionic and the electrostatic repulsive force between drops is sufficiently large to prevent coalescence, even at these low surface coverages. Note that this is particular to ionic surfactants, as emulsions and foams made with nonionic surfactants are stable only if the surface layer is dense [13, 14]. Concentrated emulsions with large drops cannot be produced using the limited coalescence process described in ref [1]: When the initial amount of surfactant is too small, the drops coalesce rapidly. The difference with Pickering emulsions is probably due to the difference in the values of *ω*, typically about 10^4^ m^−2^ s^−1^ with surfactants [15] and 10^2^ m^−2^ s^−1^ with particles [1].

If dilute emulsions are prepared in the surfactant-poor regime and not left to evolve toward the well-covered stage in the emulsifying machine, these emulsions destabilize rapidly, first by gravity: In W/O emulsions, oil separates out while water drops sediment, until the drops are in contact and gravity is not sufficient to compact them much further [13]. Once the drops are in contact, coalescence begins and water separates out. The drop compaction is larger close to the interface between the emulsion and the separated water, so coalescence mainly occurs in this region, and the drop size in the bulk emulsion remains about constant. A similar behavior is encountered with O/W emulsions [16].

**Fig. 2:**
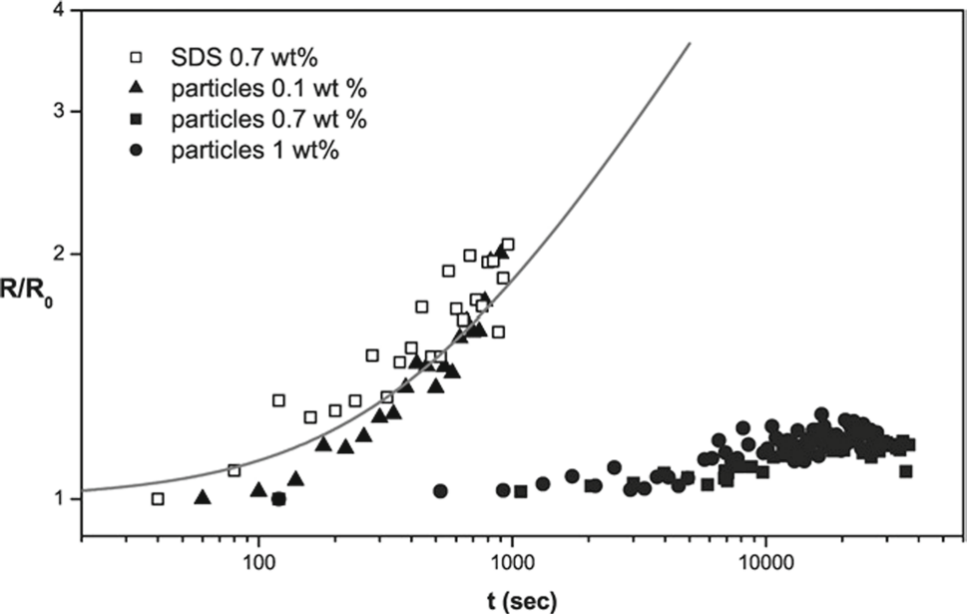
Normalized average bubble radius versus time for SDS- and particle-stabilized foams, the latter made with different bulk particle concentrations, prepared via turbulent mixing. The particles are partially hydrophobic silica. The liquid volume fraction in these foams is 0.25. The line is a fit with a square root variation of the radius versus time. Reprinted with permission from ref [17]

One could have expected to observe limited coalescence in foams stabilized by particles, sometimes called *Pickering foams*. Figure 2 shows the time evolution of the bubble radius of foams made with partially hydrophobic silica together with that for a surfactant foam.

Figure 2 shows that when the particles concentration is not enough to cover the bubbles (0.1 wt%), the foam destabilizes as rapidly as a surfactant foam. These particular foams were studied in a rotating cell in order to prevent gravity drainage [17]. However, Ostwald ripening could not be suppressed as in the emulsions of the EPJE paper, which were made using silicone oils, highly insoluble in water. In the foams, the gas present in the smallest bubbles diffuses through the aqueous films between bubbles toward larger bubbles in which the capillary pressure is smaller. The bubble radius is predicted to increase as the square root of time, as observed (see the line in Fig. 2). When the amount of particles is increased, ripening is halted: This is because the particle layer is compact and the surface elastic compression modulus becomes higher than about half the surface tension as predicted by Gibbs in order to halt Ostwald ripening [18]. More rigorous conditions have been proposed later [19], but the corrections are negligible in the foams of ref. [17]. As with the Pickering emulsions, when ripening is halted, coalescence does not occur either. One may wonder why bubble growth is not halted trough a limited coalescence process when the particle concentration is small as in emulsions. During ripening, the larger bubbles grow and their surface coverage decreases, while the smaller bubbles shrink and their surface coverage increases. The shrinking process can be halted once the surface coverage is large enough, leading to bimodal size distributions in these foams [20]. The rate of ripening in foams (*ω*_rip_ ~ 10^6^ m^−2^ s^−1^) is larger than the coalescence rates in emulsions with drop sizes similar to bubble sizes [1], likely preventing the occurrence of a limited coalescence process.

Note that in his paper, Wiley mention cases where behavior similar to limited coalescence was observed in foams [10]. In these foams, the initial bubble size was much larger than in the foams of Fig. 2: millimeters instead of 35 µm. Foam ripening becomes slower as the bubble radius R increases, R^2^ increasing linearly with time. It is possible that the ripening rate becomes smaller than the coalescence rate in foams with large bubbles.

## State of the field and future directions

The EPJE paper brought interesting information on the coalescence process, which is still far from being fully understood. It was interesting to observe that coalescence can lead to relatively narrow drop size distributions instead of broad distributions as in other coalescence processes. It was also quite interesting to show that the process is independent of the mixing type (except for the final drop size) and of the drop volume fraction, allowing the fabrication of both direct and inverse emulsion with average droplet sizes ranging from micron to millimeter. The paper was highly cited (more than 500 citations in the Web of Science, 30 in 2025), demonstrating that it has largely contributed to the progress in the field and that it is still of wide interest today.

The high resistance to coalescence is a major interest in emulsion stabilization by particles. These emulsions found applications in several fields, in particular cosmetic and pharmaceutical applications, where avoiding using surfactants is often required, due to unwanted effects such as irritation or hemolytic behavior [21]. More generally, despite their low production costs and long shelf lives, surfactants are not easily degradable and could be environmentally toxic. The increasing interest of industries in developing environmentally safe products has led to biotechnological advances involving the synthesis of green surfactants. However, the high cost of production of green surfactants still poses problems. Particles therefore offer an interesting alternative, explaining the continued interest for Pickering emulsions.

Colloidal particles are generally used, such as silica either modified chemically or through the physisorption of oppositely charged surface-active molecules [22]. Due to the ease of preparation and its applicability to very different types of particles and surface-active molecules, the latter is becoming a popular method for the production of emulsions (despite they generally make use of cationic surfactants, the less environmentally friendly type of surfactant). Other popular particles include nanocellulose [23], nanogels [24] and protein-based particles [25]. Larger particles can also be used [26]. It should be kept in mind that the amount of particles required to cover drop surfaces is proportional to the particle size and becomes large for micron-sized particles.

Pickering emulsions are encountered or purposely used in a variety of fields: oil recovery, food industry, cosmetics, pharmaceuticals, nanomedicine, soil remediation, water purification, catalysis, 3D printing, controlled combustion, coatings, templates for solid materials. Information can be found in the articles that recently cited the EPJE paper.

As discussed earlier the fundamental understanding of the underlying stabilizing mechanisms in Pickering emulsions is recent. The EPJE paper was an important step forward and will continue to inspire advances in the field.
